# Cross-Cultural Adaptation, Translation, and Validation of the Toronto Extremity Salvage Score for Extremity Bone and Soft Tissue Tumor Patients in Netherlands

**DOI:** 10.1155/2017/6197525

**Published:** 2017-07-20

**Authors:** Julie J. Willeumier, C. W. P. G. van der Wal, Robert J. P. van der Wal, P. D. S. Dijkstra, Thea P. M. Vliet Vlieland, Michiel A. J. van de Sande

**Affiliations:** Department of Orthopaedic Surgery, Leiden University Medical Center, Leiden, Netherlands

## Abstract

**Purpose:**

The aim of this study was to translate and culturally adapt the Toronto Extremity Salvage Score (TESS) to Dutch and to validate the translated version.

**Methods:**

The TESS lower and upper extremity versions (LE and UE) were translated to Dutch according to international guidelines. The translated version was validated in 98 patients with surgically treated bone or soft tissue tumors of the LE or UE. To assess test-retest reliability, participants were asked to fill in a second questionnaire after one week. Construct validity was determined by computing Spearman rank correlations with the Short Form- (SF-) 36.

**Results:**

The internal consistency (0.957 and 0.938 for LE and UE, resp.) and test-retest reliability (intraclass correlation coefficients 0.963 and 0.969 for LE and UE, resp.) were good for both questionnaires. The Dutch LE and UE TESS versions correlated most strongly with the SF-36 physical function dimension (*r* = 0.737 for LE, 0.726 for UE) and the physical component summary score (*r* = 0.811 and 0.797 for LE and UE).

**Interpretation:**

The Dutch TESS questionnaire for lower and upper extremities is a consistent, reliable, and valid instrument to measure patient-reported physical function in surgically treated patients with a soft tissue or bone tumor.

## 1. Introduction

The preferred treatment of bone and soft tissue tumors of the extremities is limb-sparing surgery. Measuring physical function after surgery is of the utmost importance to determine the success of treatment and to improve patient care. Patient-reported outcome measures enable the surgeon and the patient to objectively evaluate the patient's pain and function in order to optimize clinical care.

The Toronto Extremity Salvage Score (TESS) [1] is a valid and reliable disease-specific measure developed to evaluate physical disability in patients treated for extremity sarcoma. Different questionnaires are available for the upper and lower extremities. The TESS was originally developed in English and has currently been translated and validated in five other languages (Japanese [2, 3], Korean [4], Chinese [5], Danish [6], and Portuguese [7]).

While the TESS is commonly used in the Netherlands, it has not been translated or validated for use in the Dutch language using standardized and methodologically sound procedures. The current study aims to translate and culturally adapt the TESS (for upper and lower extremities) to Dutch and to validate the translated version among patients with surgically treated bone or soft tissue tumors of the extremities.

## 2. Methods

This research was reviewed and approved by the Medical Ethical Committee of the Leiden University Medical Center. A waiver for informed consent was provided based on the law for medical research on humans in the Netherlands (April 2016; P16.060).

### 2.1. Translation and Cross-Cultural Adaptation

The methodology used for translation and adaptation concerns a well-established process, based on published guidelines for the cross-cultural adaptation of self-reported measures by Beaton et al. [8] and Guillemin et al. [9]. During the course of translation, adaptation, and validation the TESS questionnaires for the lower extremity (LE) and upper extremity (UE) were handled separately. Forward translation from the English TESS into Dutch was performed by three bilingual translators, with Dutch as mother tongue (JJW, CWPGvdW, and JB). One of these translators (JB) was unaware of the concepts addressed and without a medical background. This led to a first Dutch consensus version. Two independent, bilingual translators with English as mother tongue and without medical background subsequently translated the Dutch version back to English (MH, TT). The expert committee, compromising a methodologist (TVV), the principal investigator (MAJvdS), and four translators (JJW, CWPGvdW, JB, and TT) reviewed all versions and components of the original questionnaire and the translations to reach consensus on the final wording to be used in the Dutch version of the TESS.

### 2.2. Patients

Consecutive eligible patients who visited the outpatient clinic between July and September 2016 (regarding LE) or February 2017 (regarding UE) for follow-up of previous surgery for bone or soft tissue tumors of the extremities were invited to complete the translated and adapted TESS. Eligible patients were identified by checking the electronic medical records of patients scheduled for follow-up. Inclusion criteria were (i) being 18 or older, (ii) a minimum of 3 months since surgical treatment for an aggressive benign or malignant bone tumor or soft tissue sarcoma, and (iii) no sign of local or systemic recurrent disease. Patients with whom communication was impaired or who could not complete questionnaires unaided were not asked to complete the questionnaires. Baseline characteristics of the participating patients, including age, gender, primary tumor, location of primary tumor, and time since primary surgery were collected.

### 2.3. Instruments

The TESS is a self-administered questionnaire that includes 30 items regarding activity limitations in daily life, such as restrictions in body movement, mobility, self-care, and performance of daily tasks and routine. The degree of physical disability is rated from 0 (not possible) to 5 (without any problem). The raw score is converted to a score ranging from 0 to 100 points, with higher scores indicating less functional limitations. Patients are able to answer questions concerning activities they do not perform in daily life with “not applicable.” These questions are deducted from the calculation of the total score.

The SF-36 is a widely used questionnaire to survey health-related quality of life [10]. The SF-36 has been validated for the Dutch population [11] and is administered as part of standard-care protocol in our hospital. The questionnaire measures eight dimensions of health and reports a score (from 0 (worst) to 100 (best)) for each category [10]. The scores from the eight categories can also be grouped into two summary scores: the physical and mental component summary scores (PCS and MCS). These summary scores were standardized using normative data from the Dutch general population with a mean score of 50 and standard deviation of 10 [11]. The scores give an indication of the functioning of the patient population in comparison with the general population.

### 2.4. Assessments

Eligible patients were invited to participate in the study by a research assistant when presenting at the outpatient clinic. The questionnaires were provided on paper. The first questionnaire was to be completed while waiting for the outpatient appointment. The second questionnaire (with a stamped return envelope) was handed out at the outpatient clinic together with the first questionnaire and patients were asked to complete the questionnaire one week later at home and send return by post. The questionnaires were paired by a code, to enable test-retest analysis.

Once patients agreed to participate in the study and their name was recorded. Patient identifying information was however not coupled to the questionnaire number, thus ensuring anonymity of the questionnaire.

### 2.5. Analyses

Prior to analysis, patients who answered 80% or more of the questions of the first TESS questionnaire with “not applicable” were excluded. For calculation of mean scores and analyses of difficult or “not applicable” questions, the first completed questionnaire of each patient was used.

### 2.6. Reliability

Internal consistency measures the homogeneity of all parts of the instrument, and was evaluated by means of calculation of Cronbach's alpha [12]. Cronbach's alpha provides a measurement of the strength of the relationship among the items of the questionnaire, with a value of >0.80 generally being considered as acceptable for scaling of the measure [13]. Test-retest variability concerns the ability of an instrument to create reproducible results when no real change has occurred for a subject. For this purpose, the intraclass correlation coefficient (ICC) was estimated between the responses to the first (test) and the second (retest) questionnaire for each item and for the total score. Bland-Altman plots were computed to visualize the absolute differences between the two assessments against the mean of the two tests to show the limits of agreement [14].

### 2.7. Validity

Construct validity measures the extent to which the scores of an instrument relate to other widely accepted measures of the same construct. For this study, construct validity of the TESS was determined by calculating the Spearman rank correlation coefficient between the TESS and the SF-36 dimension and summary scale scores.

All statistical analyses were performed with IBM SPSS version 23.0 (Armonk, NY, USA). The strength of agreement for the correlation coefficients and the ICC was defined as strong (≥0.70), moderate (>0.50 to <0.70), and weak (≤0.50) [15]. A* p *value of <0.05 was considered statistically significant.

## 3. Results

### 3.1. Translation Process

The translators and expert committee encountered no major linguistic or cross-cultural challenges during the translation and cross-cultural adaptation phase of the TESS-LE and TESS-UE questionnaires. The translation and adaptation process finally resulted in a Dutch TESS-LE and TESS-UE questionnaire, which are available in the Appendix in Supplementary Material available online at https://doi.org/10.1155/2017/6197525.

### 3.2. Patients

**Figure 1:**
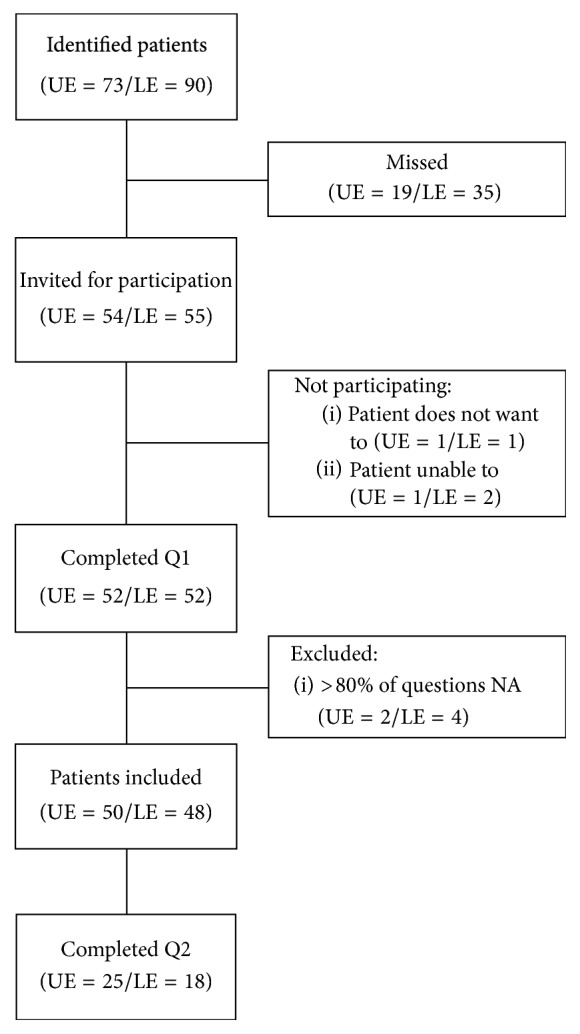
Flowchart of participating patients.

Ninety-eight patients (49% male) with a mean age of 48.7 years (range 18.1–83.8) were included (Figure 1). The characteristics of the patients and their TESS and SF-36 scores are presented in Tables 1 and 2.

### 3.3. Dutch TESS-LE and UE Questionnaire Results

Overall, the mean score of the TESS questionnaire was 77.5 (standard deviation (SD) 19.8) for the lower extremities and 90.2 (SD 14.9) for the upper extremities (Table 2). Getting up from kneeling was regarded the most difficult of all activities (mean score 3.21) in the LE questionnaire. Lifting a box to an overhead shelf was regarded the most difficult of all activities (mean score 3.94) in the UE questionnaire. Five patients (10.0%) scored a maximum score (100) on the TESS-LE, versus 19 patients (39.6%) on the TESS-UE. On the TESS-LE patients answered a median of 1 question with “not applicable” (range 0–17 questions). The questions concerning getting in and out of bath (*n* = 11, 22%), driving a car (*n* = 9, 18%), and sexual activities (*n* = 9, 18%) were most frequently answered as “not applicable.” Regarding the TESS-UE, the median number of questions answered with with “not applicable” was 0 (range 0–7 questions). The most common “not applicable” UE-activities were those about working the usual number of hours (*n* = 5, 10%) and tying a tie or bow at the neck of a blouse (*n* = 5, 10%).

### 3.4. Reliability

The internal consistency was good with Cronbach's alpha of *R* = 0.957 for the TESS-LE and *R* = 0.938 for the TESS-UE. The Spearman rank correlation coefficients between one item and the total score (excluding that item) ranged from 0.955–0.958 per item for the TESS-LE and from 0.933–0.939 per item for the TESS-UE.

**Figure 2:**
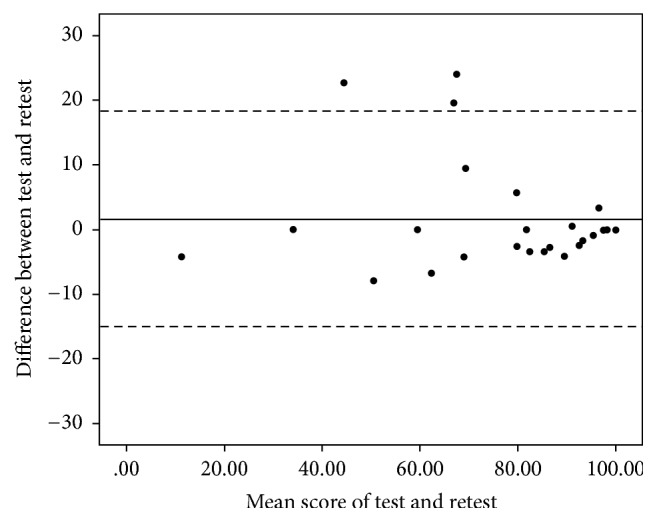
Bland-Altman plot of the test-retest reliability of the Dutch TESS-LE. The solid line shows the mean difference of the two tests (1.65) and the dashed lines show the 95% limits of agreement (−15.11; 18.41).

**Figure 3:**
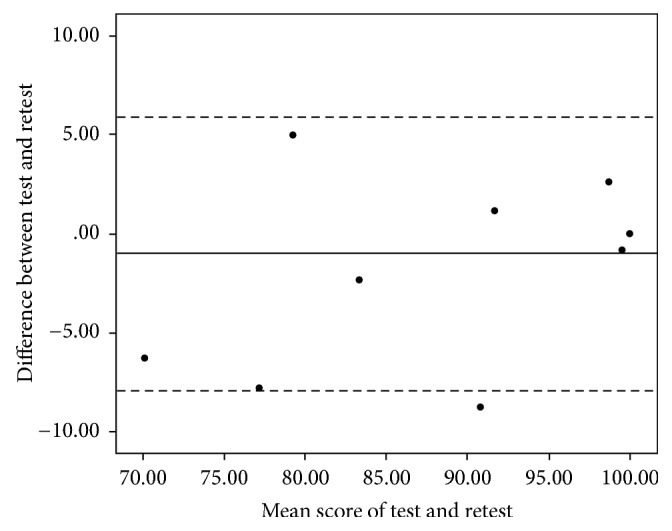
Bland-Altman plot of the test-retest reliability of the Dutch TESS-UE. The solid line shows the mean difference of the two tests (−1.01) and the dashed lines show the 95% limits of agreement (−7.89; 5.86). The dot with 0 difference between test and retest and a 100 mean score represents ten patients.

Twenty-five and eighteen of the LE (50%) and UE patients (38%) completed the “retest” questionnaire, respectively. The test-retest reliability was strong with ICC's of 0.963 (95% confidence interval (CI) 0.916–0.984) and 0.969 (95% CI 0.914–0.989) for the TESS-LE and TESS-UE, respectively. The Bland-Altman plots for both questionnaires showed there were no signs of systematic bias (Figures 2 and 3). The mean difference between the first and second questionnaire was 1.65 (SD 8.55) for the TESS-LE and −1.01 (SD 3.51) for the TESS-UE.

### 3.5. Validity

The mean scores for the eight SF-36 dimensions of the patients in the study and the physical and mental component scores (PSC/MSC) are shown in Table 2. The correlation was strong between the TESS-LE and the SF-36 dimensions physical functioning, role physical, social functioning, vitality, bodily pain, and PSC (Table 3). There was a moderate correlation between the TESS-LE and the SF-36 dimensions role emotional, mental health, and general health perceptions. The correlation with the MSC was poor. For the TESS-UE the dimensions physical functioning, role physical, bodily pain, and PSC strongly correlated, while the correlation was moderate for the dimensions social functioning, role emotional, and vitality. Mental health, general health perceptions, and MSC were poorly correlated.

## 4. Discussion

The TESS questionnaires for both the lower and upper extremities (LE and UE) are commonly used patient-reported outcome measures for functioning after the treatment of bone or soft tissue tumors in the Netherlands. However, there is currently no validated Dutch version. This study translated and culturally adapted a Dutch variant of both versions (LE and UE) of the TESS questionnaire.

The cultural adaptation was limited to a minimum, which might be due to the similarities regarding the performance of daily activities between the Canadian and the Dutch societies.

Six questionnaires were excluded from the analysis because too many (>80%) questions had been answered with “not applicable.” For both the LE and UE versions, there was one questionnaire that was completely answered with “not applicable,” of which no score could be computed. In the other four questionnaires, the number of “not applicable” answers ranged from 24 to 29. Although the summary score excludes the “not applicable” answers, a score based on only one or several items did not appear trustworthy to the authors. In the original TESS publication, no advice is given as to dealing with such outcomes neither do previous articles validating the TESS questionnaire report of questionnaires with this amount of “not applicable” answers. Reasons for the high incidence of “incomplete” questionnaires are unclear; however, the TESS was the second questionnaire to fill in, after the SF-36, and it is possible that patients ran out of patience after the first 36 questions.

The internal consistencies and test-retest reliabilities of the Dutch TESS-LE and TESS-UE were comparable with the original version of the TESS [1] and with other translated and validated versions [3–6]. As in all other versions, the test-retest reliability of the UE version was slightly higher than the LE version.

In the TESS-UE 19 patients (39.6%) scored the maximum score. This ceiling effect reduces the possibility of measuring improvement and makes discrimination in patients who are doing well difficult. In the validation of the Japanese translation of the LE-TESS a ceiling effect for 17% of the participants was registered. None of the other translation and validation studies report the presence of absence of a ceiling effect. Therefore, it is difficult to place the current result in context; was the testing group too good or is the TESS-UE really not sensitive enough to discriminate patients with good function of the upper extremity? It is however important to take this result into account when interpreting questionnaire results of individual patients with a good function.

While the original [1] and most other language versions [3–5] test the validity with the MusculoSkeletal Tumor Society (MSTS) score [16], this study tested the validity with the SF-36. The SF-36 was used as comparison with the TESS because it is standard procedure for patients to fill out the questionnaire at the outpatient clinic. Moreover, as opposed to the MSTS questionnaire which is designed as a physician-reported outcome measure, the SF-36 is designed as patient-reported outcome. From that point of view, the SF-36 is suitable to compare with the TESS, which is also patient reported. An additional comparison with the MSTS questionnaire would have brought further information, because that is a disease-specific questionnaire, but this was not possible because the MSTS questionnaire is not regularly completed by the physicians in the outpatient clinic. The correlation between the Dutch TESS (both LE and UE) and SF-36 was strong in the expected dimensions: physical component summary, physical functioning, role physical, and bodily pain. In both questionnaires the correlation with the mental component summary was poor, as was to be expected because the TESS is developed to measure physical functioning only.

This study is limited by several factors. Although the total population is sufficiently large, the subpopulations for the lower and upper extremities are small. The number of patients included in the current study was based on previous studies validating the TESS. The TESS was validated in other languages in cohorts ranging from 22 to 126 patients; thus a total of 98 patients in the current study seems reasonable. The TESS-LE was previously tested in cohorts ranging from 16 to 102 (mean 60, median 48) [3–6], so the LE cohort in this study was of average size. The TESS-UE has been validated in four other languages with small cohorts (6, 23, 43, and 56 patients). The current validation in 48 patients is thus one of the larger cohorts.

The proportion of patients returning the second questionnaire ranged between 38% and 50% which left a small group for the test-retest validity. There are no clear reasons why the return-rate was low. However, as the second questionnaire had to be filled in from home and sent by post, it is conceivable that people simply forgot. It would have been interesting to analyze whether there was a selection in the patients returning the second questionnaire. However, due to the anonymity of the questionnaires, this could not be retrieved.

The comprehension of the questions was not tested in separate questions. However, patients received verbal instructions to report any unclear questions or issues concerning the interpretation of questions to the researcher handing out the questionnaires at the outpatient clinic. Although some patients commented on the amount of questions, no issues were raised concerning the content or meaning of the questions.

The study did not test the Dutch responsiveness to the questionnaire. For use in clinical practice, especially for follow-up in the direct postoperative phase, it would have been useful to know the ability of the questionnaire to accurately detect change when this occurs. However, to test the reliability in the current validation study the population of interest was the group that was longer postoperatively and with a stable situation.

To conclude, the Dutch TESS questionnaire for UE and LE is a reliable and valid instrument to measure patient-reported physical function for patients undergoing limb salvage surgery for benign and malignant bone and soft tissue tumors. The Dutch version of the TESS can be used for future cross-cultural international studies of orthopedic oncology.

## Supplementary Material

The Dutch translation of the TESS for lower and upper extremities.

## Figures and Tables

**Table 1:** Patient and tumor characteristics of patients with benign and malignant bone and soft tissue tumors who completed the TESS questionnaire.

	TESS LE	TESS UE
*N*	50	48
Age: mean (range)	48.9 (18.6–74.9)	48.5 (18.1–83.8)
Gender: % male	47	52
Time since surgery in years: mean (range)	3.5 (0.03–18.8)	3.0 (0.03–17.8)

*Location n* (%)		
Shoulder	0	1 (2)
Humerus	0	21 (44)
Upper arm (soft tissue)	0	6 (13)
Radius	0	2 (4)
Metacarpals	0	9 (19)
Digits	0	7 (15)
Femur	22 (44)	0
Upper leg (soft tissue)	1 (2)	0
Knee	2 (4)	0
Tibia	12 (24)	0
Fibula	1 (2)	0
Lower leg (soft tissue)	3 (6)	0
Foot	2 (4)	0
Missing data^a^	7 (14)	2 (4)

*Primary tumor n* (%)		
Atypical cartilaginous tumor	10 (20)	22 (46)
Chondrosarcoma grade 2/3	5 (10)	4 (8)
Osteosarcoma	6 (12)	3 (6)
Soft tissue sarcoma	4 (8)	5 (10)
(Tenosynovial) Giant cell tumor	6 (12)	2 (4)
Osteochondroma	2 (4)	0
Fibromatosis	1 (2)	1 (2)
Cartilaginous tumour, benign	2 (2)	2 (4)
Bone other, malignant	2 (4)	1 (2)
Soft tissue other, benign	2 (4)	3 (6)
Bone other, benign	3 (6)	3 (6)
Missing data^a^	7 (14)	2 (4)

^a^Baseline characteristics were unavailable for 11 patients (7 LE and 2 UE) because they had not been recorded correctly.

**Table 2:** Mean and median scores of TESS and SF-36 for the lower and upper extremities.

	Lower extremity		Upper extremity	
	Mean (SD)	Median (range)	Mean (SD)	Median (range)
*TESS*	77.5 (19.8)	80.2 (13.3–100)	90.2 (14.9)	96.3 (21.6–100)

*SF-36*				
Physical functioning	60.5 (26.2)	65.0 (10.0–100.0)	80.4 (22.4)	85.0 (10.0–100.0)
Role limitations: physical	47.5 (43.2)	25.0 (0.0–100.0)	62.0 (42.5)	75.0 (0.0–100.0)
Social functioning	72.8 (25.3)	75.0 (0.0–100.0)	82.8 (22.6)	87.5 (12.5–100.0)
Role limitations: emotional	82.7 (33.8)	100.0 (0.0–100.0)	80.6 (36.2)	100.0 (0.0–100.0)
Mental health	72.9 (19.8)	80.0 (28.0–96.0)	78.2 (18.1)	80.0 (36.0–100.0)
Vitality	61.5 (22.6)	65.0 (15.0–100.0)	62.5 (22.3)	70.0 (15.0–100.0)
Bodily pain	62.1 (27.3)	57.1 (0.0–100.0)	72.9 (26.2)	73.5 (0.0–100.0)
General health perceptions	60.8 (25.5)	67.0 (10.0–100.0)	62.7 (19.9)	65.0 (15.0–100.0)
Physical component score	40.5 (11.2)	39.0 (16.5–58.6)	46.7 (9.9)	48.4 (23.4–61.9)
Mental component score	50.6 (10.9)	54.2 (14.0–67.9)	50.2 (9.8)	53.7 (20.5–62.8)

**Table 3:** Construct validity. Spearman rank correlations of the TESS (upper and lower extremities) with the SF-36 dimensions.

Spearman	Lower extremity	Upper extremity
Physical functioning	0.737	0.726
Role limitations: physical	0.766	0.766
Social functioning	0.810	0.585
Role limitations: emotional	0.511	0.525
Mental health	0.505	0.383
Vitality	0.704	0.586
Bodily pain	0.777	0.766
General health perceptions	0.540	0.465
Physical component score	0.811	0.797
Mental component score	0.429	0.347
