# Supplementary material for: Cross-Cultural Adaptation, Translation, and Validation of the Toronto Extremity Salvage Score for Extremity Bone and Soft Tissue Tumor Patients in Netherlands
Source: Sarcoma. 2017 Jul 20;2017:6197525. doi: 10.1155/2017/6197525 (PMC5541809; doi:10.1155/2017/6197525)
Supplement: Supplementary file 1 — The Dutch translation of the TESS for lower and upper extremities. [file 6197525.f1.pdf]

TORONTO EXTREMITY SALVAGE SCORE (TESS) – BOVENSTE EXTREMITEIT

De volgende vragen gaan over de moeite die u heeft met het uitvoeren van veel voorkomende activiteiten in het dagelijks leven. Bedenk bij elke vraag hoeveel moeite u heeft gehad met de beschreven activiteit door de klacht van uw arm gedurende **de afgelopen week**.

		Onmogelijk	Heel erg moeilijk	Moeilijk	Een beetje moeilijk	Geen enkel probleem	Niet op mij van toepassing
1	Een broek aantrekken is	1	2	3	4	5	6
2	Veters strikken is	1	2	3	4	5	6
3	Sokken of kousen aantrekken is	1	2	3	4	5	6
4	Douchen is	1	2	3	4	5	6
5	Een trui of jas aantrekken is	1	2	3	4	5	6
6	Een overhemd of blouse dichtknopen is	1	2	3	4	5	6
7	Een stropdas strikken of sjaal knopen is	1	2	3	4	5	6
8	Mijzelf opmaken of scheren is	1	2	3	4	5	6
9	Tandenpoetsen is	1	2	3	4	5	6
10	Mijn haar kammen is	1	2	3	4	5	6
11	Licht huishoudelijk werk doen is	1	2	3	4	5	6
12	Tuinieren of in de tuin werken is	1	2	3	4	5	6
13	Maaltijden klaarmaken en opdienen is	1	2	3	4	5	6
14	Eten snijden tijdens de maaltijd is:	1	2	3	4	5	6
15	Uit een glas drinken is	1	2	3	4	5	6
16	Zwaar huishoudelijk werk is	1	2	3	4	5	6
17	Winkelen is	1	2	3	4	5	6
18	Wisselgeld (munten of briefgeld) geven of ontvangen is	1	2	3	4	5	6
19	Dragen van een boodschappentas of koffertje is	1	2	3	4	5	6
20	Een doos op een plank boven het hoofd zetten is	1	2	3	4	5	6

		Onmogelijk	Heel erg moeilijk	Moeilijk	Een beetje moeilijk	Geen enkel probleem	Niet op mij van toepassing
21	Een sleutel in een slot omdraaien is	1	2	3	4	5	6
22	Een deur open duwen of trekken is	1	2	3	4	5	6
23	Schrijven is	1	2	3	4	5	6
24	Kleine voorwerpen oppakken is	1	2	3	4	5	6
25	Uitvoeren van mijn gebruikelijke taken op het werk of thuis is	1	2	3	4	5	6
26	Mijn gebruikelijke aantal uren werken op het werk of thuis is	1	2	3	4	5	6
27	Beoefenen van mijn gebruikelijke vrijetijds bestedingen is	1	2	3	4	5	6
28	Vrienden of familie bezoeken of op bezoek krijgen is	1	2	3	4	5	6
29	Mijn gebruikelijke sport- of beweegactiviteiten uitvoeren is	1	2	3	4	5	6

		Onmogelijk	Heel erg moeilijk	Moeilijk	Een beetje moeilijk	Geen enkel probleem	Niet op mij van toepassing
1	Alle activiteiten in mijn dagelijks leven in acht genomen, zou ik de moeite om deze activiteiten gedurende de afgelopen week uit te voeren omschrijven als:	1	2	3	4	5	6

		Volledig beperkt	Ernstig beperkt	Matig beperkt	Enigzins beperkt	Geheel niet beperkt
2	Ik zou mijzelf beschrijven als:	1	2	3	4	5

Gebruik onderstaande ruimte om voor u belangrijke activiteiten waarmee u moeite heeft door de klachten van uw arm, die niet in deze vragenlijst aan de orde zijn geweest, te noteren.

____

____

____

____

____

TORONTO EXTREMITY SALVAGE SCORE (TESS) – ONDERSTE EXTREMITEIT

De volgende vragen gaan over de moeite die u heeft met het uitvoeren van veel voorkomende activiteiten in het dagelijks leven. Bedenk bij elke vraag hoeveel moeite u heeft gehad met de beschreven activiteit door de klacht van uw been gedurende **de afgelopen week**.

		**Onmogelijk**	**Heel erg moeilijk**	**Moeilijk**	**Een beetje moeilijk**	**Geen enkel probleem**	**Niet op mij van toepassing**
1	Een broek aantrekken is	1	2	3	4	5	6
2	Schoenen aandoen is	1	2	3	4	5	6
3	Sokken of kousen aantrekken is	1	2	3	4	5	6
4	Douchen is	1	2	3	4	5	6
5	Licht huishoudelijk werk (zoals opruimen en stoffen) verrichten is	1	2	3	4	5	6
6	Tuinieren of werken in de tuin is	1	2	3	4	5	6
7	Maaltijden klaarmaken en opdienen is	1	2	3	4	5	6
8	Winkelen is	1	2	3	4	5	6
9	Zwaar huishoudelijk werk (zoals stofzuigen en verplaatsen van meubels) doen is	1	2	3	4	5	6
10	In en uit bad stappen is	1	2	3	4	5	6
11	Uit bed komen is	1	2	3	4	5	6
12	Opstaan uit een stoel is	1	2	3	4	5	6
13	Knielen is	1	2	3	4	5	6
14	Bukken om iets van de vloer op te pakken is	1	2	3	4	5	6
15	De trap oplopen is	1	2	3	4	5	6
16	De trap aflopen	1	2	3	4	5	6
17	Autorijden is	1	2	3	4	5	6
18	Binnenshuis lopen is	1	2	3	4	5	6
19	Buitenshuis lopen is	1	2	3	4	5	6
20	Zitten is	1	2	3	4	5	6

		Onmogelijk	Heel erg moeilijk	Moeilijk	Een beetje moeilijk	Geen enkel probleem	Niet op mij van toepassing
21	Heuvels of een (lichte) helling op of aflopen is	1	2	3	4	5	6
22	Rechtop staan is	1	2	3	4	5	6
23	Opstaan vanuit knielen of hurken is	1	2	3	4	5	6
24	In- en uit de auto stappen is	1	2	3	4	5	6
25	Seksuele activiteiten zijn	1	2	3	4	5	6
26	Uitvoeren van mijn gebruikelijke taken op het werk of thuis is	1	2	3	4	5	6
27	Mijn gebruikelijke aantal uren werken op het werk of thuis is	1	2	3	4	5	6
28	Beoefenen van mijn gebruikelijke vrijetijds bestedingen is	1	2	3	4	5	6
29	Vrienden of familie bezoeken of op bezoek krijgen is	1	2	3	4	5	6
30	Mijn gebruikelijke sport- of beweegactiviteiten uitvoeren is	1	2	3	4	5	6

		Onmogelijk	Heel erg moeilijk	Moeilijk	Een beetje moeilijk	Geen enkel probleem	Niet op mij van toepassing
1	Alle activiteiten in mijn dagelijks leven in acht genomen, zou ik de moeite om deze activiteiten gedurende de afgelopen week uit te voeren omschrijven als:	1	2	3	4	5	6

		Volledig beperkt	Ernstig beperkt	Matig beperkt	Enigzins beperkt	Geheel niet beperkt
2	Ik zou mijzelf beschrijven als:	1	2	3	4	5

Gebruik onderstaande ruimte om voor u belangrijke activiteiten waarmee u moeite heeft door de klachten van uw been, die niet in deze vragenlijst aan de orde zijn geweest, te noteren.

____

____

____
